# Electrodynamics of photo-carriers in multiferroic Eu_0.75_Y_0.25_MnO_3_

**DOI:** 10.1515/nanoph-2024-0641

**Published:** 2025-02-10

**Authors:** Yue Huang, Rolando V. Aguilar, Stuart A. Trugman, Sang-Wook Cheong, Yuan Long, Min-Cheol Lee, Jian-Xin Zhu, Priscila F.S. Rosa, Rohit P. Prasankumar, Dmitry A. Yarotski, Abul Azad, Nicholas S. Sirica, Antoinette J. Taylor

**Affiliations:** Center for Integrated Nanotechnologies, 5112 Los Alamos National Laboratory, Los Alamos, NM 87545, USA; Rutgers Center for Emergent Materials and Department of Physics and Astronomy, Rutgers University, Piscataway, NJ, 08854, USA; Enterprise Science Fund, Intellectual Ventures, Bellevue, WA, 98005, USA; U.S. Naval Research Laboratory, Washington, DC 20375, USA

**Keywords:** ultrafast spectroscopy, multiferroics, electromagnon dynamics, terahertz

## Abstract

Understanding and controlling the antiferromagnetic order in multiferroic materials on an ultrafast time scale is a long standing area of interest, due to their potential applications in spintronics and ultrafast magnetoelectric switching. We present an optical pump-terahertz (THz) probe study on multiferroic Eu_0.75_Y_0.25_MnO_3_. The optical pump predominantly excites the d-d transitions of the Mn^3+^ ions, and the temporal evolution of the pump-induced transient conductivity is measured with a subsequent THz pulse. Two distinct, temperature-dependent decay times are revealed. The shorter relaxation time corresponds to spin-lattice thermalization, while the longer one is ascribed to electron-hole recombination. A spin-selection rule in the relaxation process is proposed in the magnetic phase. Slight suppression of the electromagnons was observed after the optical pump pulse within the spin-lattice thermalization time scale. These observed fundamental magnetic processes can shed light on ultrafast control of magnetism and photoinduced phase transitions in multiferroics.

## 1 Introduction

The coexistence of electric and magnetic order in multiferroics has made these materials the subject of intense investigation, due to the possibility of indirectly manipulating charge, spin, and orbital degrees of freedom by exploiting the strong coupling between these various quantities [1], [2], [3], [4], [5]. Such coupling between ferroic orders has a clear technological appeal, as the interplay between e.g. ferroelectric and ferromagnetic order can be used to electronically (magnetically) manipulate the magnetic (electronic) state for memory storage applications [6], [7]. The relative coupling strength between these ferroic orders is dependent on their material class [8]. For type I multiferroics, the noncentrosymmetric lattice distortion responsible for driving ferroelectric order occurs independent of magnetic order, whereas many type II multiferroics show ferroelectricity that is induced by exchange striction in the magnetically ordered state. In the latter case, polar lattice distortions lower the energy of the antisymmetric Dzyaloshinskii–Moriya exchange interaction, leading to a net electric polarization that develops in the noncollinear, spiral spin state [9], [10], [11]. Such strong magnetoelectric coupling in type II multiferroics demonstrates how frustrated magnetic exchange and non-collinear spin order go hand in hand with competing exchange processes to produce ferroelectricity.

Coupling between low lying lattice and magnetic excitations in multiferroics results in a spin wave that interacts strongly with light by acquiring electric dipole activity from infrared active phonons [12], [13]. The origin of this so-called electromagnon is contested, with initial claims arguing that the same magnetoelectric coupling responsible for the emergence of ferroelectricity in the spin spiral state gives rise to the electromagnon. In this case long-wavelength fluctuations of the polarization develop in response to cycloidal spin order [7], [13]. However, selection rules predicted by this mechanism are inconsistent with polarization-dependent infrared absorption, leading to an alternate description rooted in spin-lattice coupling and shown to be fully consistent with a symmetric Heisenberg exchange model [14]. Regardless of origin, non-resonant optical excitation provides a promising route to modify electromagnons through both disrupting long-range magnetic order [15], [16], and generating photoexcited carriers capable of screening the electric polarization [17], [18]. The slight suppression and subsequent recovery of the electromagnon can be investigated on the intrinsic timescale of spin-lattice relaxation, allowing for new insights into magnetoelectric coupling.

In this manuscript, we describe optical pump – terahertz (THz) probe (OPTP) experiments on the rare-earth manganite Eu_0.75_Y_0.25_MnO_3_ (EYMO). Like other perovskite manganites (RMnO_3_; R = Gd, Dy, Tb) [19], [20], EYMO exhibits a range of magnetic and polar phases, but lacks any interference arising from the magnetic contribution of 4*f* moments [21]. Here, Jahn-Teller active, Mn^3+^ ions sit at the center of corner-sharing octahedra and undergo an orthorhombic lattice distortion brought on by the isovalent substitution of Y for Eu, enabling quasi-continuous tuning of the average rare-earth ionic radii [21]. The increased crystalline disorder brought on by such distortions leads to magnetic frustration, where a strong competition between ferromagnetic nearest neighbor (NN) and antiferromagnetic next nearest neighbor (NNN) superexchange results in collinear sinusoidal magnetic order in the ab-plane for *T* < *T*_*N*_ = 47 K [22], [23]. At *T* = 30 K, an electric polarization develops in the ac-plane (*P*_*a*_ ≫ *P*_*c*_) as a consequence of this magnetic order, leading to the emergence of two electromagnon excitations at THz frequencies (*E*_*THz*_‖*a*-axis) [13], [24]. While generally robust against an above-gap optical excitation at the *hν* = 1.55 eV resonance of Mn intersite d-d transitions, we demonstrate here a reduction of the electromagnon on a timescale consistent with spin-lattice thermalization [25], [26]. Together with the opening of a new relaxation channel in the OPTP response for *T* < *T*_*N*_, our results reveal how collective modes in this prototypical type II multiferroic compound are influenced by optical excitations possessing a characteristically larger energy scale.

## 2 Experiment and results

OPTP experiments were performed with an amplified Ti:Sapphire laser system operating at a 1 KHz repetition rate. Ultrashort optical pulses (*E* = 3.5 mJ; Δ*t* < 35 fs) centered at 800 nm (1.55 eV) were split into three beams (50:40:10) and used for pump excitation, THz generation and electro-optic detection, respectively. Broadband (0.1–2.5 THz) THz pulses generated by optical rectification in (110) ZnTe were polarized parallel to the crystallographic a-axis of a 20 μm thick (001) EYMO (*x* = 0.25) single crystal polished to <0.5 μm roughness and attached to a high-resistivity Si wafer. Transmitted THz from both EYMO as well as a high-resistivity Si reference were detected by free space electro-optic sampling using in a 0.5 mm thick ZnTe crystal, with the entirety of the THz path purged by dry air (<3%) to mitigate the effect of water absorption. Near normally incident optical pulses with a moderate pump fluence of <4 μJ/cm^2^ were used to avoid saturation in the photoinduced response, while ensuring a reasonable signal-to-noise ratio. Finally, all experiments were carried out in an optical cryostat (*T* < 10 K), allowing for the determination of the complex refractive index $\tilde{n}\left(\omega \right)=n\left(\omega \right)+ik\left(\omega \right)$ in the multiferroic phase of EYMO through use of an optical cavity model [27]:

(1) $$n\left(\omega \right)=1-\frac{c}{\omega d}\times Im\left(T_{r}\right)$$

(2) $$k\left(\omega \right)=-\frac{c}{\omega d}\left\{\ln Re\left(T_{r}\right)+\ln\frac{\left(n_{r}+n\left(\omega \right)\right)\left(1+n\left(\omega \right)\right)}{2\left(1+n_{r}\right)n\left(\omega \right)}\right\}$$

Here c is the velocity of light, *d* is the thickness of the sample, *n*_*r*_ is the refractive index of the reference, and *ω* is the frequency. Re(*T*_*r*_) and Im(*T*_*r*_) are the real and imaginary parts of the transmission, respectively.

**Figure 1:**
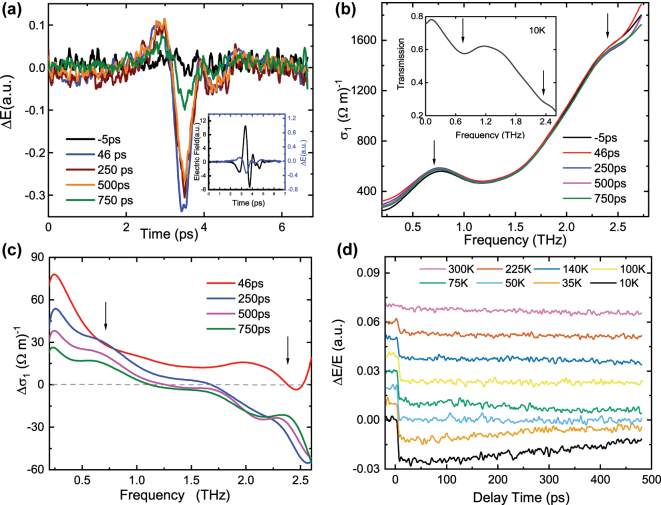
(Color online) (a) optical pump-induced change in THz field at different time delay. Inset: Time domain THz electric field transmitted through the sample without pump light (black, left axis). The difference between the THz electric field Δ*E* with and without the pump at 46 ps (blue, right axis, ×10). (b) The transient conductivity at different delay time. Inset: the transmission without pump. The arrows indicate the electromagnons. (c) The difference between conductivity with and without pump at the different delay times. All the above measurements in a,b, and c are taken at 10 K. (d) The frequency-integrated response of the 800-nm pump-THz probe signal at various temperatures: Δ*E*.

Figure 1a shows the change of the electric field Δ*E* at different delay times. Δ*E*(*t*, *t*_*p*_) is measured by placing the chopper in the optical pump beam and scanning the THz gate line. We can expect *E*_sam_(*t*, *t*_*p*_) = *E*_sam_ + Δ*E*(*t*, *t*_*p*_) [28] (*E*_sam_ is the transmitted THz electric field without optical pump), and calculate the transient transmission accordingly. The inset of Figure 1a shows the transmitted THz trace through our EYMO sample at 10 K (black, left axis) prior to pump excitation. With optical pumping, a reduction of the transmitted THz electric field by 0.2 % at Δ*t* = 46 ps can be isolated through depicting the photoinduced change Δ*E* as measured across the entire trace (blue, right axis). The complex refractive index deduced from Eq. (1) and (2) allows for both the THz transmission function and the real part of the optical conductivity, *σ*_1_(*ω*), to be computed for our EYMO sample in the multiferroic phase (T = 10 K) as a function of pump delay (Figure 1b). In the absence of pump excitation, the inset of Figure 1b reveals two local minima in the transmission at 0.7 THz (2.9 meV) and 2.4 THz (10 meV). Such features manifest as a respective peak and shoulder in *σ*_1_(*ω*), corresponding to the two electromagnon modes observed from infrared reflectivity [13]. Following optical excitation, there is an increase of spectral weight in *σ*_1_(*ω*, Δ*t*) below 1.25 THz, which progressively recedes for longer pump-probe delays. By taking the difference in the transient conductivity, Δ*σ*_1_(*ω*) = *σ*_1_(*ω*, Δ*t*) − *σ*_1_(*ω*, 0) in Figure 1c, a local reduce at the electromagnon energies is observed at a delay time of 46 ps which recovers at much longer delays (>250ps) , with the screening of the enhancement of the conductivity from photo-excited carrier at the low frequency.

Figure 1d shows the temperature dependence of the frequency-integrated pump-probe signal at the THz peak position versus delay time. For better visualization, the data at different temperatures are offset by 0.01 along the *y*-axis. The photoinduced change of transmission Δ*E*/*E* is negative which is attributed to photoexcitation of itinerant carriers following the pump excitation, and is consistent with the positive Δ*σ*_1_(*ω*) observed in Figure 1c. At high temperatures in the paramagnetic phase (*T* > 47 K), the transmission decreases abruptly following the arrival of the pump pulse, and the decrease in transmission magnitude can remain unchanged for several nanoseconds. Two additional features emerge below *T*_*N*_: the initial fast decay time increases significantly with decreasing temperature and we observe a faster recovery time of the photoinduced change in Δ*E*/*E*. We fit our differential transmission signal using bi-exponential decay function *A*_1_∗ exp(*t*/*τ*_1_) + *A*_2_∗ exp(*t*/*τ*_2_). Assuming the probe pulse has a Gaussian profile with width *γ*, the convolution of the signal and probe pulse leads to the final fit function, as described in Equation (3):

(3) $$\begin{array}{cc} \Delta E/E\left(t\right) & =C+A_{1}\exp\left(\frac{\gamma^{2}-2t\tau_{1}}{2\tau_{1}^{2}}\right)\left\{1+erf\left(\frac{-\gamma^{2}+t\tau_{1}}{\sqrt{2}\tau_{1}\gamma }\right)\right\}\\ & \;+A_{2}\exp\left(\frac{\gamma^{2}-2t\tau_{2}}{2\tau_{2}^{2}}\right)\left\{1+erf\left(\frac{-\gamma^{2}+t\tau_{2}}{\sqrt{2}\tau_{2}\gamma }\right)\right. \end{array}$$

**Figure 2:**
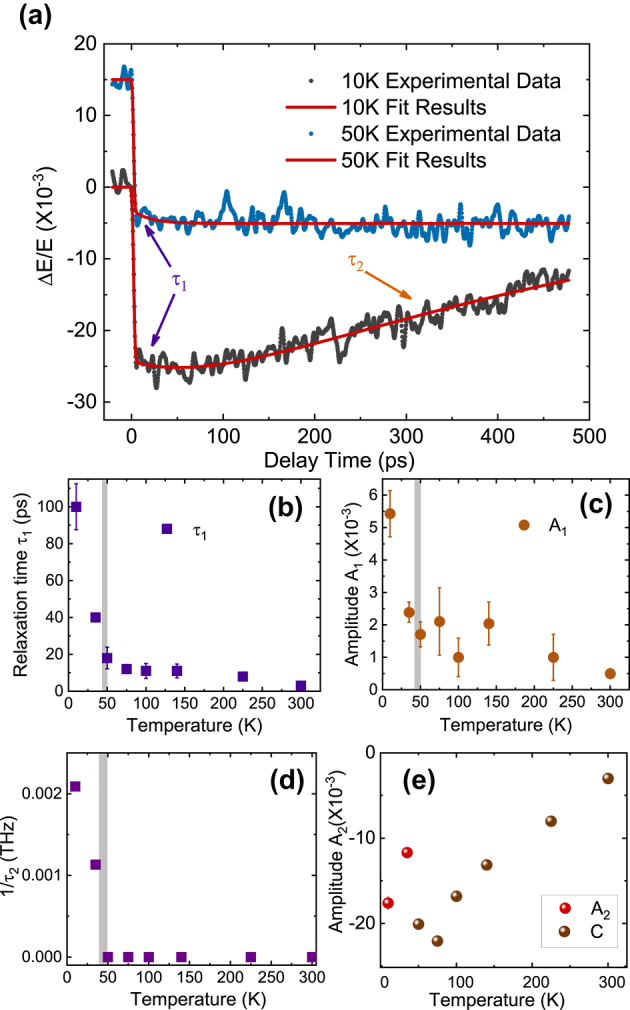
(Color online) (a) Frequency-integrated pump-probe versus delay time at 10 K and 50 K (black and blue dots; offset 0.015 along the *y*-axis from the original data). Red curve, fit to the data using the function in Eq. (3). Arrows and labels pointing to the parameters in Eq.(3): the long-rise dynamics represented by *τ*_1_ and the slow dynamics by *τ*_2_. (b) and (c) Temperature dependence of the fit parameter for *τ*_1_ and amplitude *A*_1_. (d) Temperature dependence of 1/*τ*_2_. (e) The amplitude *A*_2_ and constant *C*. For (b), (c) and (d) the grey line marks *T*_*N*_.

The first exponential term describes the initial fast decrease in Δ*E*/*E*, while the second term accounts for the recovery. The erf function is the convolution of the temporal evolution of each process with the decay and probe autocorrelation functions. In the paramagnetic phase, the recovery time extends to >10 ns, so we set *τ*_2_ to infinity in the equation by using a constant *C* instead of the second term to fit the high temperature data. The results of this fit at 10 K and 50 K are presented in Figure 2a. The initial decay time *τ*_1_ and amplitude *A*_1_, represented by the first term in equation (3), increase slowly as the temperature decreases in the paramagnetic phases and then abruptly below *T*_*N*_ (47 K), as displayed in Figure 2b and c. Next, we consider the recovery dynamics for Δ*E*/*E*. As shown in Figure 2a, the dynamics fits well to two exponential functions below *T*_*N*_, while above *T*_*N*_ the recovery extends to nanoseconds, beyond the time window of our measurement and hence we fit the dynamics with a constant *C*. We therefore plot 1/*τ*_2_ versus temperature in Figure 2d. Figure 2e shows both the amplitude *A*_2_ below *T*_*N*_ and constant *C* above *T*_*N*_.

## 3 Discussion

Excitation by the 1.55eV pump pulse creates a population of hot carriers. Subsequently, the system undergoes thermalization via electron-electron (*e* − *e*) interactions, after which the energy is exchanged among lattice, orbital, and spin degrees of freedom. Processes involving electron-electron and electron–phonon coupling occur in on a picosecond timescale and cannot be resolved using our OPTP measurements. Therefore, we do not consider these processes in our analysis.

First, we consider the process driving the initial fast decrease in the transmission of Δ*E*/*E*. We attribute this process, corresponding to *τ*_1_ and *A*_1_, to spin-lattice thermalization for the following reasons. Raman and FTIR studies on EYMO have revealed that the phonons exhibit temperature dependencies analogous to those of *τ*_1_ and *A*_1_. Raman measurements have shown that several phonons begin to soften at 100 K and continue this trend down to low temperatures [29]. FTIR measurements have revealed that the optical spectral weight of phonons shifts to lower energy as the temperature decreases [13]. In both cases, the temperature dependence is attributed to the strong spin-phonon coupling inherent to magnetic materials, and the magnitude of the change in *A*_1_ is governed by the strength of the spin correlations. Further, the previous optical pump-probe measurements on Eu_0.75_Y_0.25_MnO_3_ detected a spin-lattice relaxation time of 90 ps with a similar temperature dependence to our measurements of *τ*_1_ [25]. This spin-lattice relaxation time was also observed in other magnetic insulators such as TbMnO_3_, HoMnO_3_, and GdTiO_3_ using time-resolved THz and MOKE measurements [30], [31], [32], [33]. Below *T*_*N*_ following optical excitation, energy is transferred from the phonons to spins and the antiferromagnetic (AFM) magnetic order is disrupted, requiring a longer time for the system to return to equilibrium and a corresponding larger amplitude of the photoinduced response. Both parameters dramatically increase as the temperature is lowered. The temperature dependence and scaling of *τ*_1_ and *A*_1_ behavior are sensitive to the onset of magnetic order, suggesting that *τ*_1_ corresponds to the timescale for spin-lattice thermalization.

Next, we consider the recovery dynamics for Δ*E*/*E*, with *τ*_2_, *A*_2_, and *C* plotted in Figure 2d and e. The comparable magnitudes of *A*_2_ and *C* suggest that the same process drives the recovery in both the paramagnetic and magnetic phases, which we attribute to electron-hole (e-h) recombination. *τ*_2_ is sub-nanosecond, in the magnetic phase, and increases to >10 ns in the paramagnetic phase, consistent with the typical timescale of e-h recombination [34]. At 10 K, the transient low-frequency conductivity at 0.5 THz, which reflects the free carrier response, peaks at 46 ps and then decreases rapidly at longer delays, as shown in Figure 1c. The reduction of the low frequency conductivity spectral weight results from the lower free carrier density caused by e-h recombination. The trend of this reduction in the low-frequency spectral weight is consistent with the value of *τ*_2_. At higher temperatures, e-h thermalization can be considerably prolonged, consistent with the observed several nanosecond relaxation time. In general, the e-h relaxation does not exhibit a marked temperature dependence in semiconductors [34]. Here the pronounced correlation of the decrease of *τ*_2_ with the AFM phase transition suggests the opening of a new relaxation channel in the spin-ordered state.

**Figure 3:**
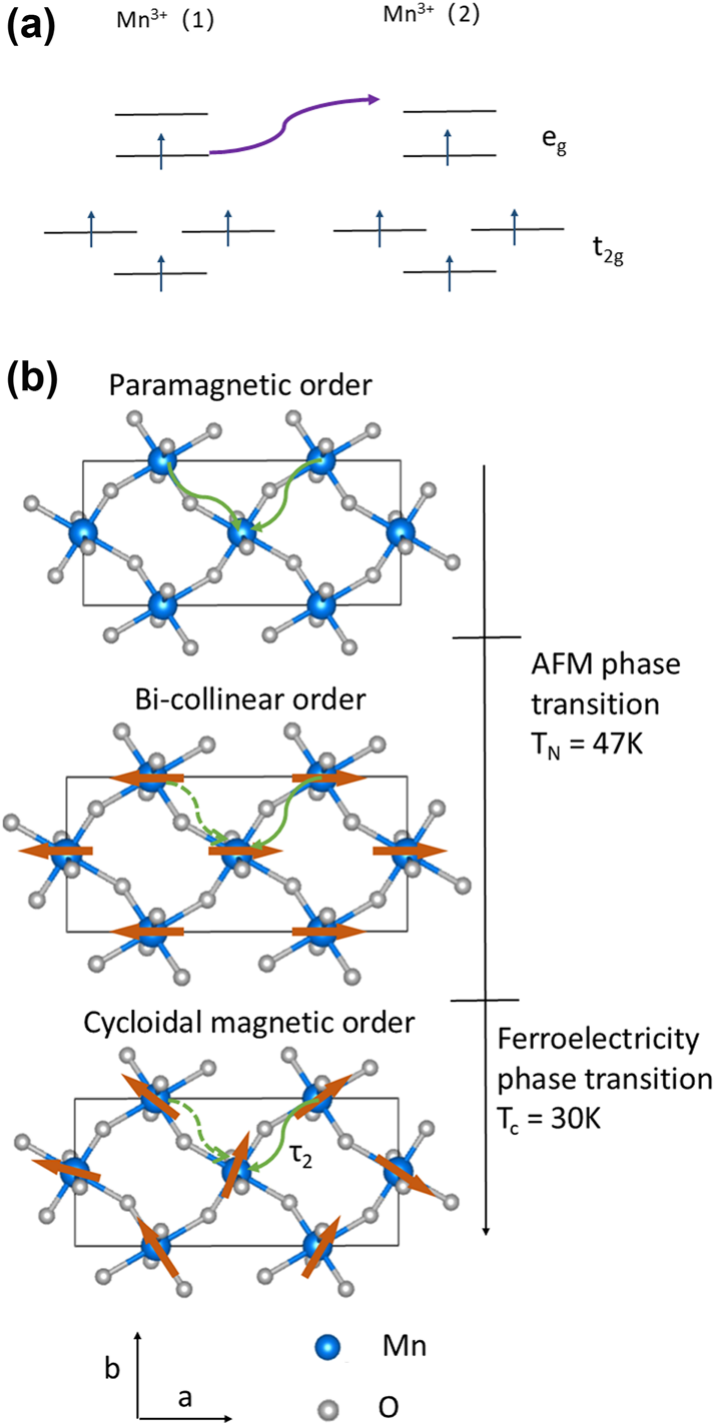
(Color online) (a) Optical transitions induced by the 800 nm pump. Absorption of a 1.55 eV photon creates a pair of *Mn* ^2+^ and *Mn* ^4+^. (b) Schematic illustration of electron and hole recombination after the 1.55 eV laser excitation in different magnetic phases (Eu and Y atoms are omitted). The orange arrow on each Mn site depicts the spin orientation. The solid arrows represents the favored recombination process and the dotted lines indicate disfavored process.

To understand the temperature dependence of *τ*_2_, we should consider the charge transfer process. The optical conductivity shows one broad absorption at 2 eV in stoichiometric rare-earth perovskite manganites, superimposed with several smaller spectral features [35], [36]. These low energy features correspond to intersite transitions of the $d_{i}^{4}d_{j}^{4}$ to $d_{i}^{3}d_{j}^{5}$ [35], [36], [37],as shown in Figure 3a. There are five possible final state configurations: a high spin (HS) state ^6^A_1_ symmetry at the energy U* − 3*J*_*H*_ + Δ_*JT*_, and four low spin states: ^4^A_1_, ^4^E_*ϵ*_, ^4^E_*θ*_, ^4^A_2_. Here U* is the effective Coulomb repulsion on the same *e*_*g*_ orbital, *J*_*H*_ is the Hund interaction, and Δ_*JT*_ is the Jahn-Teller splitting of the *e*_*g*_ levels. In contrast to the paramagnetic state, both bi-collinear or cycloidal spin alignments in the antiferromagnetic state favor HS charge transfer in the *ab* plane with similar spin orientation and suppresses them along *c* axis [35]. The electrons and holes of the HS states below *T*_*N*_ preferentially recombine with those of similar spin orientation in the *ab* plane with a higher efficiency than random recombination in the paramagnetic state. Additionally, in the AFM state, recombination between sites with different orientations such as the opposite spin in the bi-collinear magnetic state or along the *c* axis is suppressed. The recombination process is displayed in Figure 3b, with the solid line indicating the preferred process and the dotted line for unfavorable charge transfer in the different magnetic phases. This scenario explains the strong temperature dependence of *τ*_2_ we detected: the recombination relaxation time became much faster in the magnetically ordered states. We also fit the slower relaxation time (*τ*_2_) with a power law function, and obtained the similar relaxation time as that derived from the exponential function. This further supports the *τ*_2_ results from electron-holes recombination. The opening of a new channel of faster relaxation time below *T*_*N*_ is also observed in GdTiO_3_ and HoMnO_3_ compounds [30], [32], but has not been discussed in detail previously.

To delve further into the evolution of photoexcited itinerant carriers and electromagnon excitation, a Drude–Lorentz model was employed to describe the dielectric function:

(4) $$\epsilon \left(\omega \right)=\epsilon_{\infty }-\frac{\omega_{p}^{2}}{\omega^{2}+i\omega /\tau_{D}}+\overset{N}{\underset{i=1}{\sum }}\frac{S_{i}^{2}}{\omega_{i}^{2}-\omega^{2}-i\omega /\tau_{i}}$$

**Figure 4:**
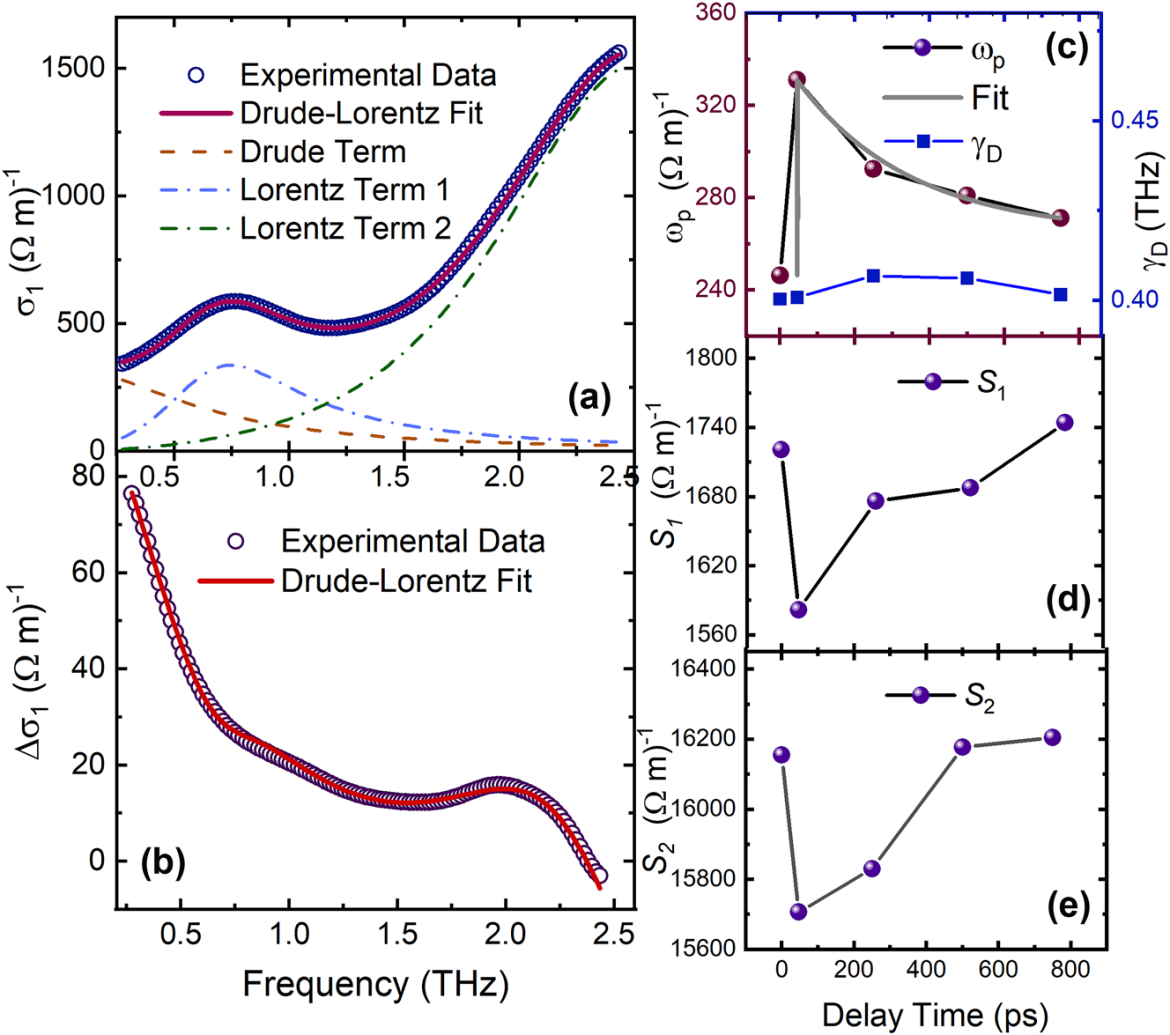
(Color online) (a) The blue circles are the transient optical real conductivity at 46 ps at 10 K. The solid curve is the Drude–Lorentz fit to the experimental data. (b) The red circles are the difference of the transient conductivity ΔΣ_1_(*t* = 46 ps) = Σ_1_(*t* = 46 ps) − Σ_1_(*t* = 0 ps). The solid curve are fit results of the experimental data. (c) Drude component of the photoexcited free carriers response: Delay time dependence of plasma frequency *ω*_*p*_ (right axis) and scattering rate *γ*_*D*_ = 1/*τ*_*D*_ at 10 K. The grey line shows the one term exponential function fit of *ω*_*p*_. (d and e) Lorentz component: delay time dependence of *S*_1_ and *S*_2_.

The first term is the Drude component depicting the free carrier response, while the second term is the Lorentz components characterizing the electromagnon. *ϵ*_*∞*_ is the dielectric constant at high frequencies. *ω*_*p*_ is the plasma frequency and *τ*_*D*_ (1/*γ*_*D*_) is the free carrier lifetime. *S*_*i*_ is the square root of the oscillator strength and the *ω*_*i*_ is the resonance frequency of Lorentz components. The model was found to reproduce the conductivity spectrum, as shown in Figure 4a and b. All fitting parameters are displayed in Table 1.

**Table 1:** The plasma frequency *ω*_*p*_ (Ω^−1^m^−1^) and scattering rate *γ*_*D*_ = 1/*τ*_*D*_ (THz) for the Drude term, the resonance frequency *ω*_*i*_ (THz), the width *γ*_*i*_ = 1/*τ*_*i*_ (THz) and the square root of the oscillator strength *S*_*i*_ (Ω^−1^m^−1^) for the Lorentz component for different time delays.

Time	*ω*_*p*_	*γ*_*D*_	*ω*_1_	*γ*_1_	*S*_1_	*ω*_2_	*γ*_2_	*S*_2_
−5 ps	246	0.4	0.74	0.471	1,721	2.46	1.05	16,154
46 ps	328	0.401	0.75	0.470	1,581	2.44	1.03	15,706
250 ps	296	0.406	0.75	0.471	1,676	2.46	1.06	15,830
500 ps	278	0.406	0.74	0.470	1,687	2.47	1.073	16,177
750 ps	272	0.401	0.74	0.472	1,744	2.48	1.076	16,205

Figure 4c shows the fit parameters of plasma frequency *ω*_*p*_ (left axis) and scattering rate (right axis) of the Drude term versus time delay. *ω*_*p*_ rises initially following optical excitation, and then decreases with the delay time. According to the Drude model, the square of *ω*_*p*_ is proportional to the effective carrier density *n*/m* (where m* is the effective carrier mass). The photoexcited charge carrier density is calculated as 1.1 × 10^−5^ per unit cell through the above relationship. Alternatively, the carrier density can also be estimated through the penetration depth. Considering a illumination of 1.55 eV light with a fluence of *F* = 4 μJ/cm^2^, the penetration depth is 6.7 × 10^−7^ m according to the absorption spectrum of EYMO [24], and the charge carrier density is calculated as 5.8 × 10^−5^ per unit cell. The estimate of carrier density from the penetration depth is larger than the value from the Drude model, because only free carriers possessing high mobility contribute to the Drude response, whereas carriers with low mobility only contribute to the penetration depth. The ratio of these two values is around 0.2, suggesting that the carriers are excited into multiple bands and only a small fraction of them are photoexcited in the high mobility band. The transition at 1.5 eV occurs between the lower Hubbard band below the Fermi level and the upper Hubbard band. The conduction bands are more complex than a simple single-band mobility. In contrast to *ω*_*p*_, the scattering rate, *γ*_*D*_, does not exhibit a strong time dependence, indicating that the scattering rate remains unaffected as the electrons relax back to the valence band. Additionally, *γ*_*D*_ is significantly slower than in conventional semiconductors. This reduced scattering rate could result from the magnetic order of the material, which suppresses scattering pathways, or it could be attributed to the electronic band into which the electrons are excited. Specifically, the upper Hubbard band in doped EYMO may contribute to this behavior due to its intrinsic electronic structure.

The plasma frequency decreases after 100 ps, showing a similar relaxation trend to *τ*_2_ shown in Figure 4c. The fit of the decay of plasma frequency versus time with an exponential function results in a relaxation time of 350 ps which is comparable to the *τ*_2_ at 10 K. Since the square of plasma frequency is proportional to the free carrier density, its decay shows a reduction of photo-excited carriers which is consistent with the e-h recombination rate, further supporting the interpretation of the recovery process corresponding to *τ*_2_ as carrier recombination.

The two Lorentz terms in Eq.(4) capture the two electromagnon modes. For the lower-frequency electromagnon, the resonance frequency *ω*_1_ is 0.74 THz corresponding to the electromagnon position reported previously [13], [24]. The peak width *γ*_1_, 0.47 THz, describes the scattering rate of the excitation. Both parameters remain essentially constant following 800-nm pump excitation. However, *S*_1_ drops by 8 % (as compared to the 1 % error level from the fitting process), and then recovers to the initial level after 200 ps, as shown in Figure 4d. The electromagnon at higher frequency shows a similar decay time dependence in Figure 4e, but the fit results may be affected by the phonon at 120 cm^−1^ (3.5 THz) which was reported in the previous optical conductivity study [13].

The oscillator strength $S_{i}^{2}$ links to the magnitude of the excitation, indicating the two electromagnon modes are affected. The *e*_*g*_ − *e*_*g*_ hopping transfer arising from the 800-nm pump, will lead to a spin frustration which does not flip the spin (not observed in our experiments). This frustration would perturb the Hamiltonian corresponding to the electromagnon excitation, resulting in a weak suppression in the amplitude of the electromagnon excitation that can be detected as a decrease in *S*_*i*_, but not a change in the electromagnon frequency, *ω*_*i*_, consistent with our measurement. The reduction of the electromagnon modes occurs within the 100 ps spin-lattice relaxation time scale. Considering the strong Heisenberg exchange model [14], the AC electric field causes distortions of the Mn–O–Mn bond configuration based on the spin-lattice coupling. Meanwhile, the relaxation time to the unperturbed state of *S*_1_ is <100 ps, consistent with the observation of the reduction at electromagnon frequencies in the transient conductivity only at 46 ps in Figure 1c. Therefore, the above strong Heisenberg exchange model explained our discovery that the timescale of the electronmagons suppression is within spin-lattice relaxation process.

## 4 Conclusions

To conclude, we performed an optical pump THz probe study on Eu_0.75_Y_0.25_MnO_3_. Our measurements found two clear relaxation times: a sub-100 ps rise time due to spin-lattice relaxation and a much longer magnetic order-related recovery due to electron-hole recombination. The spin-lattice thermalization shows a power law relationship with temperature. The temperature dependence of the electron-hole recombination suggests a channel opening below the Neel temperature, shortening the recovery time. A spin-selection rule in the relaxation process was proposed. Given its significance in the ultrafast manipulation of magnetic materials, further investigation of this process is warranted. The slight suppression of the electromagnon only found within the spin-lattice thermalization time range is consistent with the origin of electromagnon based on a strong Heisenberg exchange model. Our measurements demonstrate an attractive method for tracking the dynamics of material properties by directly probing low energy excitations such as electromagnons, and offer a powerful approach for understanding the coupling between different degrees of freedom in strongly correlated materials.
